# A machine learning approach to predict positive coronary artery calcium scores in individuals with diabetes: a cross-sectional analysis of ELSA-Brasil baseline data

**DOI:** 10.1590/1414-431X2025e14986

**Published:** 2025-08-22

**Authors:** J.L. Amorim, I.M. Bensenor, A.P. Alencar, A.C. Pereira, A.C. Goulart, P.A. Lotufo, I.S. Santos

**Affiliations:** 1 Centro de Pesquisa Clínica e Epidemiológica, Hospital Universitário, Universidade de São Paulo, São Paulo, SP, Brasil; 2 Departamento de Clínica Médica, Faculdade de Medicina, Universidade de São Paulo, São Paulo, SP, Brasil; 3 Departamento de Estatística, Instituto de Matemática e Estatística, Universidade de São Paulo, São Paulo, SP, Brasil; 4 Laboratório de Biologia Molecular, Instituto do Coração, Hospital das Clínicas, São Paulo, SP, Brazil; 5 Departamento de Epidemiologia, Faculdade de Saúde Pública, Universidade de São Paulo, São Paulo, SP, Brasil

**Keywords:** Atherosclerotic cardiovascular disease prediction, Diabetes, Coronary artery calcification, Machine learning, Supervised learning

## Abstract

It is unclear who benefits the most from atherosclerotic cardiovascular disease (ASCVD) screening imaging. This study aimed to identify features associated with positive coronary artery calcium scores (CACS) in individuals with diabetes using machine learning (ML) techniques. ELSA-Brasil is a cohort study with 15,105 participants aged 35 to 74 years in six Brazilian cities. We analyzed 25 sociodemographic, medical history, symptom-related, and laboratory variables from 585 participants from the São Paulo investigation center with CACS data and no overt cardiovascular disease at baseline. We used six ML algorithms to build models to identify individuals with positive CACS. Feature importance was determined by SHapley Additive exPlanations (SHAP) values. The best performer ML algorithm was the XGBoost Classifier (accuracy: 94.8%). Age (SHAP: 0.220), systolic blood pressure (SHAP: 0.102), and body mass index (SHAP: 0.075) were the most important variables to identify ASCVD in individuals with diabetes in XGBoost models. Considering all ML models in our analysis, age, systolic blood pressure, and sex were frequently influential variables. We obtained high accuracy with our best model, using information generally present in current clinical practice. ML models may help clinicians select patients with characteristics most probably associated with a positive CAC. Age, systolic blood pressure, body mass index, and sex may be useful markers to identify those at higher risk for subclinical ASCVD.

## Introduction

Cardiovascular diseases (CVD) are the leading cause of mortality in most countries (1). Data from the Global Burden of Disease study show an increase in the absolute number of deaths, years of life lost, and years of life adjusted for CVD disability in recent decades (2,3). CVDs are also the leading cause of death in Brazil, where age-adjusted mortality rates are still higher than in high-income countries.

Most CVD burden is due to atherosclerotic CVD (ASCVD). Diabetes is a well-known ASCVD risk factor. Persons with diabetes are more likely to develop ASCVD (4), including coronary heart disease (CHD), stroke, peripheral arterial disease, cardiomyopathy, and congestive heart failure. Before the onset of clinical conditions, ASCVD may be detected by imaging techniques such as carotid ultrasound or computed tomography (CT) for coronary artery calcium scores (CACS) quantification. Diabetes has also been associated with these markers of subclinical atherosclerosis (5,6).

Although there is consensus about the association between diabetes and ASCVD, it is unclear which subgroup of individuals with diabetes should be referred for further evaluation of atherosclerotic disease. While blood pressure measurements and resting electrocardiograms (ECGs) are recommended for most patients with diabetes (4,7), the screening for coronary artery disease (CAD) is not recommended routinely by the American Diabetes Association (ADA) in asymptomatic individuals (4). ADA recommends that CAD be investigated only in symptomatic patients (including those with atypical cardiac symptoms), signs or symptoms of associated vascular disease (including carotid bruits, transient ischemic attack, stroke, claudication, or peripheral arterial disease), or electrocardiogram abnormalities (4). On the other hand, the European Society of Cardiology (ESC) recommends that carotid or femoral ultrasound or coronary CT angiography be considered risk modifiers (7). However, the value of these advanced imaging techniques in routine practice has not yet been demonstrated.

Screening for asymptomatic CAD in patients with diabetes mellitus (DM) remains controversial. With computed tomography (CT), non-invasive estimation of the atherosclerotic burden (based on CACS) and identification of atherosclerotic plaques causing significant coronary stenosis [CT coronary angiography (CTCA)] can be performed. While a CACS of 0 is associated with a favorable prognosis in asymptomatic subjects with DM, subsequent strata in CACS (to 1-99, 100-399, and ≥400) are associated with a 25 to 33% higher relative mortality risk compared to the previous step (6).

The Brazilian Longitudinal Study of Adult Health (ELSA-Brasil) is a cohort study with 15,105 participants aged between 35 and 74 years in six centers in different cities. At baseline assessment, multiple laboratory tests were performed to diagnose diabetes. Additionally, a subset of 4,548 participants from the São Paulo investigation site underwent CACS determination (8). This created a favorable scenario to analyze which characteristics are the most important to identify individuals with diabetes with a higher probability of presenting positive CACS.

In this study, we used ML algorithms to evaluate the most important sociodemographic, clinical, and laboratory features to predict positive CACS in individuals with diabetes at ELSA-Brasil baseline assessment.

## Material and Methods

### Study design

This study used data from the ELSA-Brasil cohort (8), a prospective study focused on diabetes, cardiovascular, and other related chronic diseases of the Brazilian population (9). The study baseline was between 2008 and 2010, with 15,105 participants aged between 35 and 74 years in six centers in different cities in the country (Belo Horizonte, Porto Alegre, Rio de Janeiro, Salvador, São Paulo, and Vitória). The largest ELSA-Brasil research center is in São Paulo, with 5,061 participants (10). In the São Paulo investigation center, participants were invited to assess CACS by computed tomography at baseline.

### Study sample

The São Paulo investigation site had 5,061 participants, from which 4,548 (89.9% from the São Paulo set) had their coronary artery calcium scores (CACS) determined. In this set, we identified 649 individuals with diabetes. Finally, after excluding 64 (9.9%) individuals with previous myocardial infarction (MI), stroke, or myocardial revascularization, we defined the remaining 585 as our study sample.

### Diabetes definition

The diagnosis of diabetes at baseline of the ELSA-Brasil was based on the presence of at least one of the following criteria: medical history of diabetes, use of medications for diabetes treatment, fasting glucose ≥126 mg/dL, oral glucose tolerance test after 2 h ≥200 mg/dL or glycated hemoglobin ≥6.5% (9).

### CACS assessment

The scans were performed with a 64-detector CT scanner (Philips Brilliance; Philips, Netherlands). The field of view included the entire heart, and the axis direction included data from the bifurcation of the pulmonary arteries to the apex of the heart during an expiratory pause. The images were analyzed using specific software (Brilliance Workspace). Data are reported as the absolute value of CACS in Agatston points (10,11). Higher CACS are associated with more advanced atherosclerosis. The presence of coronary artery calcification, as indicative of subclinical ASCVD, was defined as a CACS>0.

### Other variables

Initial variable selection from the ELSA-Brasil dataset followed targeted meaningful clinical information. Therefore, we preselected variables containing information usually retrieved during the first consultations of an individual with diabetes in standard clinical practice. Age, sex, educational level, income, race, smoking status, family history of CVD, prior hypertension or dyslipidemia diagnosis, time from diagnosis, and medication use were self-reported. Hypertension was defined as using medication to treat hypertension, systolic blood pressure ≥140 mmHg, or diastolic blood pressure ≥90 mmHg. High-density lipoprotein (HDL), triglycerides, and total cholesterol were included. Dyslipidemia was defined as low-density lipoprotein (LDL)-cholesterol ≥130 mg/dL or the use of cholesterol lowering medications. Glycated hemoglobin, blood glucose, and body mass index were also included. Chronic kidney disease (CKD) was defined as glomerular filtration rate (GFR) <60 mL/min/1.73 m^2^ using the CKD-Epi formula (Chronic Kidney Disease Epidemiology Collaboration) (12), as previously published about CKD in ELSA-Brasil (13,14).

According to the Rose questionnaire, dyspnea was classified according to intensity as: 1) no dyspnea, 2) dyspnea during intense activity, 3) dyspnea during light activity, and 4) dyspnea at rest. Additionally, chest pain was classified as: 1) no chest pain, 2) atypical chest pain, 3) chest pain when walking fast, and 4) chest pain when walking slowly (Supplementary Data S1) (15,16). The complete list of variables used is described in Supplementary Table S1.

### Statistical analysis

Data management is detailed in Supplementary Data S2. Categorical variables are presented as counts and proportions and continuous variables are presented as means and standard deviations. We used extreme gradient boosting (XGBoost), random forest (RF), K-nearest neighbor (KNN), logistic regression (LR), support vector machine (SVM), and decision tree (DT) ML techniques (17-22) to classify study participants according to subclinical/undiagnosed ASCVD status. The ML algorithms selected for analyses were extensively tested in different research scenarios. Supplementary Tables S2 to S7 present the parameter sets for all models. Explanatory variables were sociodemographic (age, sex, race, educational level), medical history (hypertension and dyslipidemia treatment and time since diagnoses, smoking status, and family history of CVD), symptom-related (recurrent exertional chest pain or dyspnea at baseline), clinical (body-mass index, systolic and diastolic blood pressure), and laboratory (fasting and post-load plasma glucose, glycated hemoglobin, total, LDL, and HDL cholesterol levels, triglycerides, and estimated glomerular filtration rate). Data were randomly divided into training (n=468; 80%), validation (n=59; 10,1%), and test (n=58; 9,9%) sets, following best practice procedures (23-25). Model accuracy and related metrics were calculated using the test set. Feature importance was determined by SHapley Additive exPlanations (SHAP) values (26). SHAP values evaluate which variables are the most influential in model prediction and are applied to various ML techniques under a unified framework. According to this metric, we present the five most influential variables in each model. Although the decision tree model had lower accuracy than other models, we also show the first three decision nodes in this model, as its results are easy to interpret. We used Python language version 3.10 and the ML library scikit-learn 1.3.0 to produce this work.

## Results

Table 1 presents the characteristics of the study sample. There were 294 participants with a CACS=0 (53.1% women) and 291 participants with a CACS>0 (64.9% men). The average age was 56 years (interquartile range [IQR]: 49-62 years). High age, male sex, low glomerular filtration rate (all P<0.001), high albumin-creatinine ratio, White race (P=0.006 for both), dyslipidemia (P=0.042), and current smoking (P=0.044) were associated with CACS>0.

**Table 1:** Characteristics of the study sample according to coronary artery calcium score (CACS) values.

Variable	CACS=0 (n=294)	CACS>0 (n=291)	Total (n=585)
Male sex (%)	138 (46.9%)	189 (64.9%)	327 (55.9%)
Age (years)	52.6±8.4	59.1±8.4	55.8±9.0
Self-reported race (%)			
Black	61 (20.7%)	34 (11.7%)	95 (16.2%)
Mixed	78 (26.5%)	8 (23.4%)	146 (25.0%)
White	144 (49.0%)	152 (52.2%)	296 (50.6%)
Other	11 (3.7%)	37 (12.7%)	48 (8.2%)
Educational level (%)			
Less than high school	65 (22.1%)	73 (25.1%)	138 (23.6%)
High school	138 (46.9%)	91 (31.3%)	229 (39.1%)
College or above	91 (31.0%)	127 (43.6%)	218 (37.3%)
Income (%)			
Low (<USD$1245)	119 (40.5%)	80 (27.5%)	199 (34.0%)
Middle (USD$1245-3319)	120 (40.8%)	121 (41.6%)	241 (41.2%)
High (≥USD$3320)	55 (18.7%)	90 (30.9%)	145 (24.8)
Smoking (%)			
Never smoked	165 (56.1%)	127 (43.6%)	292 (49.9%)
Past smoker	94 (32.0%)	122 (41.9%)	216 (36.9%)
Current smoker	35 (11.9%)	42 (14.4%)	77 (13.2%)
Hypertension (%)	179 (60.9%)	208 (71.5%)	387 (66.2%)
Dyslipidemia (%)	138 (46.9%)	182 (62.5%)	320 (54.7%)
Use of antihypertensives (%)	157 (53.4%)	179 (61.5%)	336 (57.4%)
Use of blood glucose lowering medication (%)	157 (53.4%)	195 (67.0%)	352 (60.2%)
Use of lipid-lowering medication (%)	215 (73.1%)	183 (62.9%)	398 (68.0%)
Dyspnea (%)			
No dyspnea	213 (72.4%)	246 (84.5%)	459 (78.5%)
During intense activities	69 (23.5%)	38 (13.1%)	107 (18.3%)
During light activities	8 (2.7%)	3 (1.0%)	11 (1.9%)
At rest	4 (1.4%)	4 (1.4%)	8 (1.4%)
Chest pain (%)			
No chest pain	253 (86.1%)	255 (87.6%)	508 (86.8%)
Atypical	19 (6.5%)	24 (8.2%)	43 (7.4%)
Walking fast	16 (5.4%)	9 (3.1%)	25 (4.3%)
Walking slow	6 (2.0%)	3 (1.0%)	9 (1.5%)
Body mass index (kg/m^2^)	30.6±5.4	29.4±4.4	30.0±5.0
Systolic blood pressure (mmHg)	124.8±17.0	130.5±18.5	127.6±18.0
Diastolic blood pressure (mmHg)	78.7±10.9	79.3±10.9	79.0±10.9
Fasting plasma glucose (mg/dL)	140.2±51.8	151.0±57.8	145.6±55.0
Glycated hemoglobin (%)	6.5±1.5	6.7±1.7	6.6±1.6
HDL cholesterol (mg/dL)	49.5±11.5	49.2±11.8	49.3±11.6
LDL cholesterol (mg/dL)	110.5±36.6	114.0±38.6	112.3±37.6
Triglycerides (mg/dL)	169.0±130.2	156.6±84.6	162.8±110.0
Total cholesterol (mg/dL)	195.1±46.9	197.8±52.0	196.5±49.5
Serum creatinine (mg/dL)	0.92±0.2	1.0±0.4	0.96±0.3
Albumin-creatinine ratio (mg/g)	18.6±62.7	71.3±383.6	44.8±275.2
Glomerular filtration rate (mL/min/1.73 m^2^)	83.7±15.3	78.2±17.1	80.9±16.4

The glomerular filtration rate was calculated according to the CKD-Epi estimation equation.

Table 2 presents the performance metrics for the models used in this study. Model accuracy varied from 67.2% (decision trees) to 94.8% (XGB). In this model, age (SHAP: 0.220), systolic blood pressure (SHAP: 0.102), and body mass index (SHAP: 0.075) were the variables with the highest SHAP values. The list of the five variables with the highest SHAP values for all models is shown in Table 3. Age (all models), systolic blood pressure, and sex (five models) were the most frequent variables of the most influential variables. More detailed SHAP value information for all ML models is presented in Supplementary Table S8 and Supplementary Figure S1.

**Table 2:** Performance metrics for the machine learning models.

Machine learning model	CACS	Performance metric			
		Precision	Recall	F1-score	Accuracy
X Gradient Boosting	CACS=0	0.92	0.98	0.95	0.95
	CACS>0	0.98	0.92	0.95	
Random Forest	CACS=0	0.94	0.85	0.89	0.91
	CACS>0	0.88	0.95	0.92	
K-Nearest Neighbors	CACS=0	0.94	0.83	0.88	0.89
	CACS>0	0.84	0.95	0.89	
Logistic Regression	CACS=0	0.71	0.69	0.70	0.73
	CACS>0	0.75	0.77	0.76	
Support Vector Machines	CACS=0	0.69	0.66	0.67	0.69
	CACS>0	0.69	0.72	0.70	
Decision Tree	CACS=0	0.62	0.74	0.68	0.67
	CACS>0	0.73	0.61	0.67	

CACS: coronary artery calcium score. Metrics definitions are as follows: Precision=(TP) / (TP + FP); Recall = (TP) / (TP + FN); F1-Score = 2 * [(Precision * Recall) / (Precision + Recall)]; Accuracy = (TP + TN) / (TP + FP + FN + TN). TP: true positive; TN: true negative; FP: false positive; FN: false negative.

**Table 3:** SHapley Additive exPlanations (SHAP) values for the five most influent variables in each machine learning model.

Variable	SHAP
X Gradient Boosting	
Age	22.0
Systolic blood pressure	10.2
Body Mass Index	7.5
Albumin-Creatinine Ratio	7.2
Blood Glucose	6.5
Logistic Regression	
Age	65.7
Sex	56.3
Use of blood glucose lowering medication	33.8
Dyslipidemia	32.5
Black race	30.2
Random Forest	
Age	27.6
Systolic blood pressure	6.7
Sex	5.8
Blood glucose	4.7
Glomerular filtration rate	4.2
Support Vector Machines	
Age	25.7
Sex	15.7
Dyslipidemia	7.5
Dyspnea during intense activity	5.7
Systolic blood pressure	5.5
K-Nearest Neighbors	
Age	21.0
Blood glucose	12.8
Systolic blood pressure	10.0
Glomerular filtration rate	7.4
Albumin-creatinine ratio	6.4
Decision Tree	
Age	44.2
Systolic blood pressure	5.9
Sex	4.7
Blood glucose	4.6
Glomerular filtration rate	0.1

SHAP values are reported in 10^-2^ units.

**Figure 1:**
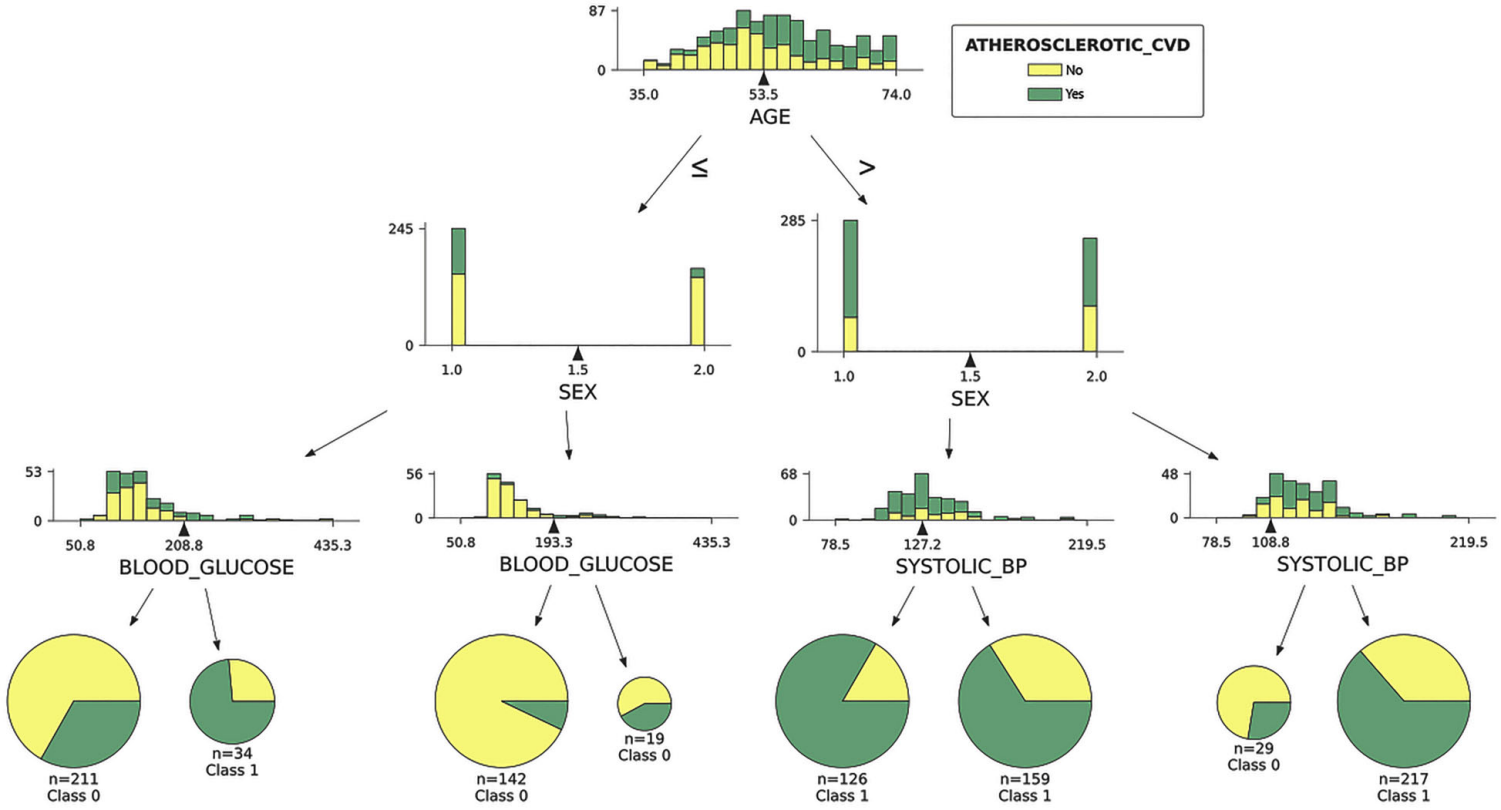
Decision tree model for positive coronary artery calcium (CAC) classification. CVD: cardiovascular disease; BP: blood pressure; Class 0: CAC=0; Class 1: CAC>0.

Due to the easy and intuitive interpretation of decision tree results, we also show the three first decision nodes in that model (Figure 1). Age and sex were the main variables for predicting positive CACS in decision tree models. With a cutoff at 53.5 years, blood glucose (young men and women) and systolic blood pressure (older men and women) were the variables selected in the initial nodes.

## Discussion

In our study, model accuracy for predicting positive CACS in ELSA-Brasil participants with ASCVD varied across ML models from 67.2 to 94.8%. The algorithm with the best performance was the XGBoost, and in that model, the most influential variables in identifying individuals with positive CACS were age, systolic blood pressure, and BMI. Considering all ML models in our analysis, age, systolic blood pressure, and sex were frequent influential variables. This reinforces the intertwined relationship between diabetes and other cardiovascular risk factors in the development of ASCVD.

Developing models that accurately identify ASCVD may result in better resource allocation and reduced CVD burden. This is even more promising if these models include routine healthcare data, reducing extra costs. Other authors have used ML models to predict ASCVD and related conditions in different settings. Miranda et al. (27) used a dataset including age, sex, and hematological test data from 6,837 individuals, 4,702 of whom had CAD. Their best model (adaptive boosting) had an accuracy of 78% in identifying individuals with CAD diagnosis. Although those authors also reported SHAP values (the highest absolute values were attributed to sex, leukocyte count, and thrombocyte count), it is important to note that they did not include variables clinically related to ASCVD, such as high blood pressure, blood glucose, or smoking. Given the correlation between blood cell counts and some traditional ASCVD risk factors (28), this may impair the interpretation of variable importance in those analyses. Miao et al. (29) used adaptive boosting performance to identify individuals with CAD in four medical-based datasets. They found accuracy rates from 77.8% (Long Beach Medical Center) to 96.7% (University Hospital in Switzerland). The very high accuracy found for the latter may be partially explained by imbalanced data, as 93.4% of individuals in that dataset had CAD diagnosis. Lee et al. analyzed data from 2,133 individuals from Korea who underwent coronary CT during a health checkup program to identify whether LR, XGBoost, or CatBoost (Categorical Boost Classifier) were accurate in detecting participants who had had a CACS>100. Input features included age, sex, and anthropometric and laboratory exams. The best performance was achieved by XGBoost (area under the receiver operating characteristic [AUROC] curve: 0.82), followed by catboost (AUROC: 0.75) and LR (AUROC: 0.59) (30). In comparison, the ensemble models in our study (RF and XGBoost) and KNN showed at least as high accuracies as these studies. Based on those results, we may hypothesize that ML models may support the decision to investigate subclinical or undiagnosed ASCVD in individuals with diabetes. However, it is important to note that intervention studies are needed to quantify the risks and benefits of this screening strategy before it is applied clinical practice.

In our article, age, sex, and systolic blood pressure were important features for classification. They were the top 3 influential variables (according to SHAP values) in two ML models (RF and DT). They were frequent among the other models' top 5 influential variables. It is noteworthy that systolic blood pressure was at least as informative for classification in our study as diabetes control (measured by fasting plasma glucose and glycated hemoglobin). Blood pressure (BP), glycated hemoglobin, and LDL-cholesterol contribute to CHD risk among patients with diabetes, and most clinical guidelines address these risk factors, aiming at strict control goals (31). Although a recent study by Wong et al. (32) including pooled data from three large cohorts in the US suggests that BP control may be a minor contributor to CHD risk in individuals with diabetes (compared to glycated hemoglobin and LDL-cholesterol), our data pointed otherwise. We may hypothesize two reasons for this finding in our study. Wong et al. studied CHD incidence in individuals with diabetes over a period of approximately 10 years while we evaluated the presence of coronary calcification. Although CHD events and subclinical atherosclerosis arguably represent a continuum of the same phenomenon, cardiovascular risk factors may play different roles in triggering CHD events (for example, leading to plaque instability and rupture) (33,34), which at least partially explain the observed differences. Second, this may be a population-specific finding, and blood pressure control can be a more important determinant of atherosclerotic disease in the Brazilian population with diabetes, reinforcing the need to explore this association in our population. Finally, our data also suggest that one should not rely too heavily on the absence of cardiovascular symptoms, such as dyspnea and chest pain, to rule out ASCVD in individuals with diabetes, as these variables had little influence in the most accurate models.

Our study has strengths. ELSA-Brasil is a large epidemiological study with documented protocols and quality control (35). This ensures data integrity through high-quality clinical data to address relevant clinical conditions, as in the present study. In our study, we could address subclinical atherosclerosis using coronary CT. Further studies are needed to establish CACS potential for CVD screening and the optimal target population for coronary CT, while helping enhance CVD risk estimation in specific settings. It is reasonable to argue that the most promising of these settings is the quantification of atherosclerosis development in individuals at high presumable CVD risk, such as those with diabetes and additional risk factors. Some limitations must also be pointed out. As we could not apply the selected models in external samples, it is not possible to draw conclusions about the accuracy of our models in other populations. Therefore, some of our findings may be specific to our population. Our sample size was relatively small, mainly because the analyses had to be restricted only to participants from the ELSA-Brasil investigation site in São Paulo. On the other hand, hosting this study in ELSA-Brasil ensured high-quality data and data integrity (as explained above). Additionally, we had a very balanced dataset (according to the response variable), which optimized analyses and minimized problems due to sample size.

In conclusion, we built highly accurate ML models to identify individuals with diabetes with positive CACS at the ELSA-Brasil baseline. The algorithm with the best performance was XGBoost. Considering all ML models, the most influential variables in classification were age, systolic blood pressure, and sex. These findings may support clinical decisions when assessing if an individual with diabetes should be referred for CACS assessment. Future studies may address whether CVD screening using coronary CT in patients classified at higher risk according to ML-based models is beneficial and cost-effective.

## Supplementary Material

Click to view [pdf].
