# Supplementary material for: A machine learning approach to predict positive coronary artery calcium scores in individuals with diabetes: a cross-sectional analysis of ELSA-Brasil baseline data
Source: Braz J Med Biol Res. 2025 Aug 22;58:e14986. doi: 10.1590/1414-431X2025e14986 (PMC12377704; doi:10.1590/1414-431X2025e14986)

Data S1. Collection and scoring of dyspnea, chest pain, and angina pectoris variables.

Dyspnea
Can you climb 8 stairway steps carrying weight (±10 kg), walk fast on flat terrain, or go uphill without having to stop because of tiredness or breathlessness?
Can you walk slowly on the ground level, tidy up the house or take a bath without stopping because of tiredness or breathlessness?
Do you have tiredness or breathlessness while eating, sitting or lying down?

Level	Description	Value
DYSPNEA_NONE	No dyspnea	0
DYSPNEA_INTENSE_ACT	Dyspnea during intense activities	1
DYSPNEA_LIGHT_ACT	Dyspnea during light activities	2
DYSPNEA_IN_REST	Dyspnea at rest	3

Chest Pain
Have you experienced chest pain or discomfort?
Do you feel this chest pain while walking on ground level?
Do you feel this chest pain when you climb a ladder, or a stairway, or walk fast on flat terrain?

Variable	Description	Value
CHEST_PAIN_NONE	No chest pain	0
CHEST_PAIN_ATYPICAL	Atypical chest pain	1
CHEST_PAIN_WALK_FAST	Chest pain walking fast	2
CHEST_PAIN_WALK_SLOW	Chest pain walking slow	3

Table S1. List of variables potentially included in machine learning models.

AGE	Age
HYPERTENSION	Hypertension diagnosis
DYSLIPIDEMIA	Dyslipidemia diagnosis
ANTIHYPERTENSIVES	Use of antihypertensives
HYPOGLYCEMIANTS	Use of blood glucose lowering medication
LIPID_LOWERING	Use of lipid-lowering medication
BODY_MASS_INDEX	Body-mass index
SYSTOLIC_BP	Systolic blood pressure
DIASTOLIC_BP	Diastolic blood pressure
BLOOD_GLUCOSE	Blood glucose
GLYCATED_HEMOGLOBIN	Glycated hemoglobin
HDL	HDL cholesterol
LDL	LDL cholesterol
TRIGLYCERIDES	Triglycerides
TOTAL_CHOLESTEROL	Total cholesterol
CREATININE	Serum creatinine
ALBUMIN_CREATININE	Albumin-Creatinine Ratio
GLOMERULAR_FILTRATION	Glomerular filtration rate (CKDEpi)
SEX	Sex
RACE_BLACK	Black race
RACE_BROWN	Brown race
RACE_WHITE	White race
RACE_OTHERS	Other races
EDUCATION_ELEMENTARY	Up to incomplete high school
EDUCATION_SECONDARY	High school
EDUCATION_UNIVERSITY	College education or above
INCOME_LOW	Low income (<US$ 1245)
INCOME_MIDDLE	Middle income (US$ 1245-3319)
INCOME_HIGH	High income (≥ US$ 3320)
SMOKING_NEVER	Never smoker
SMOKING_PAST	Past smoker
SMOKING_PRESENT	Current smoker
DYSPNEA_NONE	No dyspnea
DYSPNEA_INTENSE_ACT	Dyspnea during intense activities
DYSPNEA_LIGHT_ACT	Dyspnea during light activities
DYSPNEA_IN_REST	Dyspnea at rest
CHEST_PAIN_NONE	No chest pain
CHEST_PAIN_ATYPICAL	Atypical chest pain
CHEST_PAIN_WALK_FAST	Chest pain walking fast
CHEST_PAIN_WALK_SLOW	Chest pain walking slow
ATHEROSCLEROTIC_CVD	Positive coronary artery calcium*

*Positive coronary artery calcium is the dependent variable.

Data S2. The dataset was handled according to data engineering procedures (data cleaning, data checking, variable recodification and variable transformation). We performed an exploratory data analysis (EDA) to check descriptive statistics and select, create new, and delete unnecessary patient variables due to high correlation (see heatmap plots below). After this process, the 15 variables with the highest SHAP values were maintained in the model.

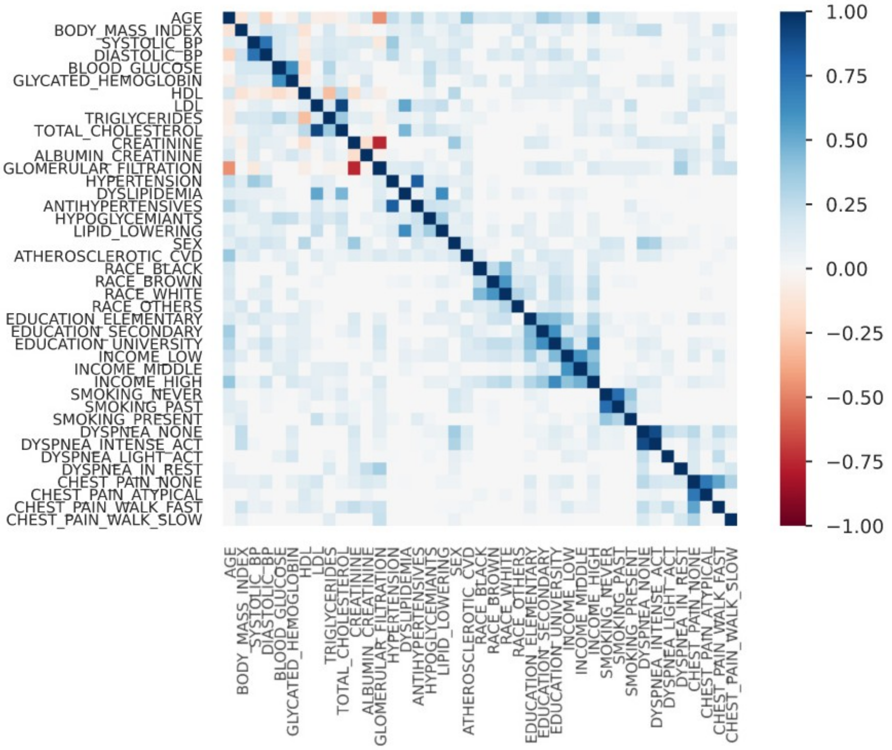

Table S2. Setup parameters for X Gradient Boosting models.

Hyperparameter	Value
base_score	0.5
booster	gbtree
callbacks	None
colsample_bylevel	1
colsample_bynode	1
colsample_bytree	1
early_stopping_rounds	None
enable_categorical	False
eval_metric	None
feature_types	None
gamma	0
gpu_id	-1
grow_policy	depthwise
importance_type	None
interaction_constraints	'',
learning_rate	1
max_bin	256
max_cat_threshold	64
max_cat_to_onehot	4
max_delta_step	0
max_depth	6
max_leaves	0
min_child_weight	1
missing	nan
monotone_constraints	'()',
n_estimators	500
n_jobs	0
num_parallel_tree	1
predictor	auto
random_state	42

Table S3. Setup parameters for random forests classifiers.

Hyperparameter	Value
bootstrap	True
ccp_alpha	0.0
class_weight	None
criterion	gini
max_depth	10
max_features	10
max_leaf_nodes	None
max_samples	None
min_impurity_decrease	0.0
min_samples_leaf	1
min_samples_split	2
min_weight_fraction_leaf	0.0
n_estimators	200
n_jobs	None
oob_score	False
random_state	42
verbose	0
warm_start	False

Table S4. Setup parameters for K-nearest neighbors classifier.

Hyperparameter	Value
algorithm	kd_tree
leaf_size	20
metric	minkowski
metric_params	None
n_jobs	None
n_neighbors	30
p	2
weights	distance

Table S5. Setup parameters for logistic regression models.

Hyperparameter	Value
C	1.5
class_weight	None
dual	False
fit_intercept	True
intercept_scaling	1
l1_ratio	None
max_iter	20000
multi_class	auto
n_jobs	None
penalty	l2
random_state	None
solver	liblinear
tol	0.0001
verbose	0
warm_start	False

Table S6. Setup parameters for decision trees

Hyperparameter	Value
ccp_alpha	0.0
class_weight	None
criterion	entropy
max_depth	3
max_features	None
max_leaf_nodes	None
min_impurity_decrease	0.0
min_samples_leaf	1
min_samples_split	2
min_weight_fraction_leaf	0.0
random_state	42
splitter	best

Table S7. Setup parameters for support vector machines.

**Hyperparameter**	**Value**
C	1.0
break_ties	False
cache_size	200
class_weight	None
coef0	0.0
decision_function_shape	ovr
degree	3
gamma	scale
kernel	linear
max_iter	-1
probability	False
random_state	42
shrinking	True
tol	0.001
verbose	False

Table S8. Feature importance based on SHapley Additive exPlanations (SHAP) values.

Model	Variable	SHAP value ($\times 10^2$)
X Gradient Boosting	Age	22.0
	Systolic blood pressure	10.2
	Body mass index	7.5
	Albumin-creatinine ratio	7.2
	Blood glucose	6.5
	Glomerular filtration rate	5.9
Random Forest	Age	31.7
	Systolic blood pressure	8.0
	Sex	5.1
	Blood glucose	4.8
	Glomerular filtration rate	4.2
	Body mass index	4.1
K-Nearest Neighbors	Age	21.1
	Blood glucose	12.8
	Systolic blood pressure	10.0
	Glomerular filtration rate	7.4
	Albumin-creatinine ratio	6.4
	Body mass index	4.2
Logistic Regression	Age	52.3
	Sex	41.1
	Use of blood glucose lowering medication	32.6
	Dyslipidemia	28.5
	Black race	21.3
	Other races	15.1
	Body mass index	14.0
	Dyspnea during intense activities	13.1
	Systolic blood pressure	12.8
	Glomerular filtration rate	10.4
	High income	9.1
	Blood glucose	8.9
	Smoking past	8.2
Support Vector Machines	Age	25.7
	Sex	15.7
	Dyslipidemia	7.5
	Dyspnea during intense activities	5.7
	Systolic blood pressure	5.5
	Past smoker	5.5
	Use of blood glucose lowering medication	5.1
	Blood glucose	4.7
	Black race	4.5
	Glomerular filtration rate	4.2
	Other races	3.4
	Body mass index	2.3
	Hypertension diagnosis	0.9
Decision Tree	Age	44.2
	Systolic blood pressure	5.9
	Sex	4.7
	Blood glucose	4.6
	Glomerular filtration rate	0.0

Figure S1. SHapley Additive exPlanations (SHAP) values for machine learning models.

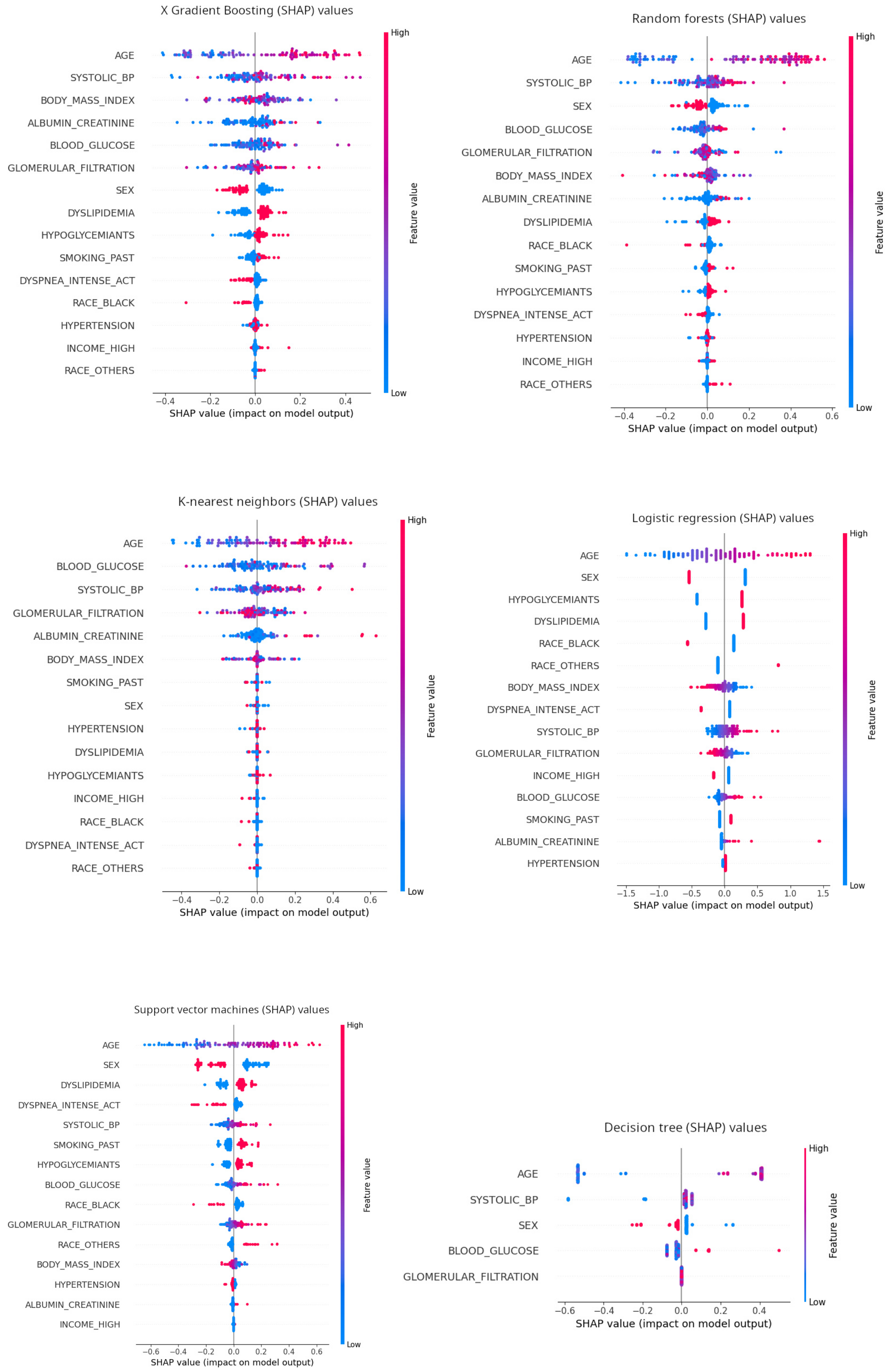

Supplement: Supplementary file 1 [file 1414-431X-bjmbr-58-e14986-suppl.pdf]
